# To Hunt or Patrol? Social Composition and Location Mediate Scent Marking Decisions of a Large Carnivore

**DOI:** 10.1002/ece3.71567

**Published:** 2025-06-18

**Authors:** K. Whitney Hansen, Neil R. Jordan, Megan J. Claase, J. W. McNutt, Alan Wilson, Christopher C. Wilmers

**Affiliations:** ^1^ Environmental Studies Department University of California Santa Cruz California USA; ^2^ Botswana Predator Conservation Maun Botswana; ^3^ Center for Ecosystem Science, School of BEES University of New South Wales Sydney Australia; ^4^ Taronga Conservation Society Australia Dubbo New South Wales UK; ^5^ Structure & Motion Lab, Comparative Biomedical Sciences Royal Veterinary College London UK

**Keywords:** behavioral trade‐offs, *Lycaon pictus*, resource selection analysis, scent marking, sociality, territoriality

## Abstract

While sociality is known to mediate territorial processes, it is less clear how sociality interacts with environmental features and neighbors' location to influence habitat selection and behavior. Scent marking, a fundamental behavior in maintaining territories, can be utilized by receiving conspecifics to evaluate both encounter risk and competitive ability of the depositing individual or group. African wild dog packs were followed in the field across 2010–2021, where researchers recorded individual behaviors and pack composition, including scent marking behaviors. We combined this historical and unique behavioral dataset with co‐occurring GPS collar data to make inferences on territorial behaviors, sociality, and habitat selection across spatial scales. We performed three analyses to determine (1) the relative probability of scent mark placement, (2) the probability of scent marking, and (3) the trade‐off strategy between scent marking and hunting, as predicted by habitat, neighbors' territories, and pack social composition. Specifically, we used resource selection function frameworks to determine how and whether conspecifics influenced habitat selection and behavior at multiple orders of selection. We found that conspecifics were influential across all three analyses, and mediated the impact of habitat on scent mark placement and probability. Scent mark placement and probability were both influenced by the social composition of packs, specifically pup presence, pack size, and number of overlapping neighbors, while pack size and pack experience influenced territorial maintenance strategy. Our findings demonstrate the importance of social structure across scales of territorial processes, from larger scale habitat selection to the probability of a behavior. We demonstrate how key behavioral theories underpinning territoriality function at the scale of habitat selection and behavioral decision‐making in a free‐ranging, large carnivore. Future research should continue to incorporate sociality in understanding the habitat selection of animals.

## Graphical abstract

Using a unique set of near‐continuous behavioral observations of free‐ranging wild dog packs, we combine field data with GPS locations to make inferences on how sociality mediates territorial strategies in a large carnivore. By investigating habitat selection across territorial behaviors, we make multi‐scale inferences on how inter‐ and intra‐pack structures mediate territorial maintenance.

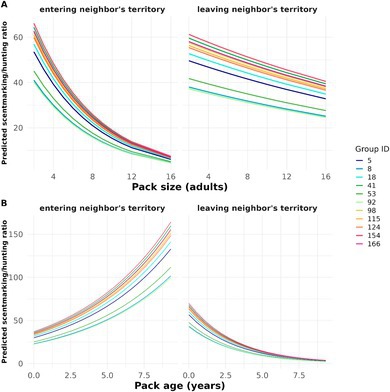

## 1 Introduction

Territoriality in vertebrate species is a widespread phenomenon, and species have different proximate causes for establishing and maintaining territories (Maher and Lott 2000). While the ultimate causes of territoriality stem from population density and reproductive fitness, proximate causes of territoriality are managed by aggression and energetic constraints (Brown 1964). Individuals produce signs of presence (i.e., scent marks) around or within their territory to communicate the threat of defensive action without incurring costs from physical interactions with neighbors (Gosling 1982). Scent marking has been shown to signal territory ownership or occupancy (Lewis and Moorcroft 2001), advertise to mates (Zala et al. 2004), and communicate to neighbors (Claase et al. 2024). So where and when individuals scent mark, and how they respond to others' scent marks, illuminates how an individual perceives and uses space (Macdonald 1980; Gosling 1982). However, signaling is only an evolutionary stable strategy when signalers elicit responses from receivers that are mutually beneficial (Davies et al. 2012). For example, territory owners advertise their presence to monopolize resources, while conspecifics benefit by using its information to avoid physical combat with resource holders (Lewis and Moorcroft 2001). By matching deposited scent to certain individuals or groups (e.g., scent matching), conspecifics can dually assess the likelihood of meeting and escalation (Gosling and McKay 1990). Therefore, the spatial distribution of scent marks reflects a communication strategy on the part of the sender (scent marker) shaped by the target receiver (Gosling and Roberts 2001).

Empirical evidence has shown that the consequences of territorial spatial structures extend to individuals' movement on a fine scale (Young and Monfort 2009; Moriarty et al. 2017). These fine scale choices influencing physical interactions with neighbors, or signals of their presence, directly impact an individual's territory establishment and maintenance, which translates to broader‐level population processes (e.g., population size, offspring survival, litter size, see Pitt et al. 2003). When it comes to behavioral trade‐off's in territorial maintenance, theoretical models have explored when to defend a territory based on the potential benefits of its exclusive (or near‐exclusive) access by the owner (Switzer et al. 2001; Morrell and Kokko 2005; Hinsch and Komdeur 2010). The trade‐off between available resources from a territory, and the cost of patrolling said territory, is a known energetic trade‐off in how animals maintain their territory (Ord 2021). It also serves as an indicator of that animal's individual social status (such as a dispersing vs. resident individual, or overall reproductive quality) (Gosling and Roberts 2001). A component of what makes territories more or less difficult to maintain is the rate of territorial intrusion, which impacts optimal territory size (Schoener 1987). Territoriality is the spatial demonstration of social hierarchies in populations (Kaufmann 1983), and therefore different types of individuals, or groups, will have the social dominance to either perpetrate or better withstand more territorial intrusions (Potts et al. 2019). Therefore, when considering how energetic trade‐off's in territorial maintenance affect space use in animals, it is equally vital to consider how the social landscape of a population will mediate may process. We know that the social composition within (e.g., reproductive status and age of group members) or among (e.g., relatedness or relative size to adjacent groups) territorial groups mediates territorial processes, such as avoidance or selection of neighbors' territorial space (Hansen et al. 2024), overlap with neighbors' territories (Jackson et al. 2017), territory size (Bateman et al. 2014), and territorial formation (Ellison et al. 2020). Further work, however, is needed to consider how social composition (within or among groups) mediates individual behavioral decisions that sum to territorial maintenance and place scent marking behavior within the context of space use and habitat selection more broadly.

Scent marking theory suggests that animals benefit from intruders encountering their scent and assessing their competitive ability (Gosling and McKay 1990), implying that scent marking placement is a crucial component of territorial function and in the management of direct encounters and conflict. It has been theoretically demonstrated that competing individuals make decisions to engage in conflict or not based on cumulative experience and fighting ability (Hamilton and McNutt 1997), and therefore social information gained from scent could be a powerful mechanism in animal decision‐making. Scent functions as an investment by the owner in establishing their territory, which affects the likelihood of fight escalation given that the owner has more to lose than the intruder has to gain (Gosling and McKay 1990). A combination of economic constraints (e.g., time and energy needed to deposit scent), resource heterogeneity (e.g., how clustered defensible resources are), and territory composition (e.g., size) all interact to influence scent mark placement among species and ecosystems (Gosling 1982). Canids, for instance, are a highly social family and often use scent to communicate (Macdonald and Sillero‐Zubiri 2004). The physical distributions and strategies of scent signaling in canids have some consistent features across species, specifically selection for linear features (such as roads and crossroads) to place scent (Ralls and Smith 2004; Stępniak et al. 2020), selecting slightly raised/elevated ground (Barja and List 2014; Sillero‐Zubiri and Macdonald 1998), the use of specific checkpoints (such as latrines) (Buesching and Jordan 2019), and social bias in scent marking intensity (Gosling and Roberts 2001; Jordan et al. 2014). However, scent mark placement in particular can vary: packs of Ethiopian wolves scent mark along boundaries (Sillero‐Zubiri and Macdonald 1998) while other species, such as the African wild dog ( *Lycaon pictus* ), do so throughout the territory in a “cloud” (Parker 2010). Additionally, even within‐species individuals vary in scent mark placement based on their population, habitat availability, and/or an individual's social status such as sex, age, or competitive ability (Macdonald 1980).

Sociality in general is an underappreciated mechanism driving movement and behavioral decisions in wildlife (Webber et al. 2022), and while recent work has contributed important insights into broader‐scale space use patterns and habitat selection (Jesmer et al. 2018; Prokopenko et al. 2024; Hansen et al. 2024), we do not yet fully understand how social mechanisms permeate to finer scale behaviors on the landscape. Behavioral responses of individuals or groups to invading neighbors, specifically with regards to the “nasty neighbor” or “dear enemy” hypotheses, are influenced by both the social identity of groups and territorial location of invasion (Temeles 1994; Müller and Manser 2007). These two hypotheses propose alternate explanations for invading neighbors; the nasty neighbor hypothesis presumes that animals reserve aggressive responses for known conspecifics they need to maintain dominance over, while the dear enemy hypothesis suggests animals reduce aggression toward known entities and instead target unknown newcomers (Temeles 1994; Müller and Manser 2007). However, neither hypothesis truly considers how responses might change according to perceived social dominance of known neighbors, and research suggests that group size affects how groups treat their known neighbors (Claase et al. 2024). Subsequently, we do not know whether or how the social structure of groups alter behavioral trade‐off strategies when it comes to scent marking strategy, nor how this affects subsequent space use.

The African wild dog is a territorial carnivore forming large packs of an average of 10.4 individuals, with some packs comprised of up to 30 individuals (Creel et al. 2004). Packs are typically comprised of one reproductively active pair of adults (the dominants) supported by their offspring which are born into the pack during an annual denning season (McNutt 1996; Creel et al. 2004). Through their system of scent communication (Claase et al. 2022), packs maintain large territories (739 ± 81 km^2^) (Creel et al. 2004; Pomilia et al. 2015; Jordan et al. 2023) and avoid direct confrontation with conspecifics from neighboring packs (Jordan et al. 2017; Chisholm et al. 2019) despite significant territorial overlap (Pomilia et al. 2015). This high degree of territorial overlap leads to fluid territorial dynamics that vary across levels of relative social dominance (Hansen et al. 2024), and a socially‐dependent, fine scale communication strategy that heavily relies on scent marking (Claase et al. 2024). Several social attributes such as kinship, pack size, pack experience, pup presence, and breeding status all mediate territorial interactions (Creel et al. 2004; Jackson et al. 2017; Hansen et al. 2024). Additionally, dominant scent marks elicit a more pronounced response by fellow pack members (Jordan et al. 2013) and other packs (Claase et al. 2024) than do scents from subdominants. However, further work is needed to distill the adaptive significance of scent mark placement in the African wild dog and other large carnivores (Apps et al. 2022; Rafiq et al. 2020). Additionally, while the presence of conspecifics has been shown to influence scent mark placement and territorial layouts (Lewis and Moorcroft 2001), it is less clear how intra‐group sociality (e.g., relative group sizes or ages) mediates scent mark placement. Scent provides important information on competitive ability (Gosling and McKay 1990) which, based on predictions from the Ideal Despotic Distribution (Calsbeek and Sinervo 2002), suggests that the combination of its placement and the socially‐relevant information it provides should scale to influence habitat selection and space use of individuals on a larger scale (Hansen et al. 2024). Given the complex social dynamics among packs and importance of scent marking in territorial maintenance, the African wild dog is well suited to the study of how sociality mediates behavioral decision‐making in territorial processes.

This study investigates scent marking behavior in a large carnivore across spatiotemporal scales, within different social contexts, and relative to other behaviors. We linked sociality, behavior, and space use in order to unravel how socially mediated, behavioral decisions scale to territorial maintenance in the African wild dog. First, we sought to understand how neighbors influence scent marking behavior relative to habitat selection. Second, we determined how packs select where to scent mark relative to other behaviors on a fine scale, specifically considering neighbors and social dynamics in addition to habitat features. Finally, we investigate how the relative location of neighbors and group composition affect behavioral trade‐off strategies between hunting and scent marking on the landscape. Specifically, we predicted that the relative territorial layouts would mediate how packs select scent marking locations, but that packs should still prioritize marking in areas that maximize exposure to neighbors: open habitat and roads, and boundary areas where there is greater overlap between packs (e.g., contested zones) (Roberts and Lowen 1997; Sillero‐Zubiri and Macdonald 1998; Barja and List 2014; Allen et al. 2017; Rafiq et al. 2020; Stępniak et al. 2020). Second, we predicted that the social structure of a pack should mediate what behaviors packs employ when in areas with increasing neighbor presence: more vulnerable packs (packs with pups, smaller packs, and younger/less experienced packs) should be less likely to advertise presence than their counterparts (Hansen et al. 2024; Claase et al. 2024). Lastly, we predicted that we would see the culmination of scent marking decisions vis‐à‐vis a behavioral trade‐off between hunting and scent marking at an aggregate level, where moving packs would be more likely to scent mark than hunt in neighboring territory. However, we also hypothesized that this trade‐off behavior would be mediated by pack size and experience. By combining behavioral theory with movement ecology inference, this study sheds light on the social drivers of behavior and space use.

## 2 Materials and Methods

### 2.1 Study Area

This study took place in the southwestern Okavango Delta of Botswana (study site is ca. 2600 km^2^; 19°31′ S, 23°37′ E; elevation ca. 950 m). The study area is composed of multiple habitat types, mainly floodplains, grasslands, savannah woodlands, and shrublands, some of which vary seasonally according to the Delta's flooding schedule (McNutt 1996). The Delta's rainy season lasts from December to March, while the early flood season lasts from April to July and culminates in a peak flooding season from August to November, due to the downflow of rains that fell higher up the catchment in the preceding wet season (Bennitt et al. 2014). The seasonally flooded wetland of the Delta supports a rich diversity of ungulate species (Rich et al. 2016). While these species include larger species such as the Cape buffalo ( *Syncerus caffer* ), blue wildebeest ( *Connochaetes taurinus* ), and Burchell's zebra ( *Equus quagga* ), wild dogs preferentially hunt medium‐sized antelope species, such as the impala ( *Aepyceros melampus* ), steenbok ( *Raphicerus campestris* ), and bush duikers ( *Sylvicapra grimmia* ) (Tshimologo et al. 2021). Larger packs also target the greater kudu ( *Tragelaphus strepsiceros* ) and tsessebe ( *Damaliscus lunatus* ) (Tshimologo et al. 2021). The Okavango Delta comprises the heart of the Southern African region, which supports the largest contiguous remaining population of African wild dogs (KAZA TFCA Secretariat 2014). Multiple private safari operators are based in the study area, and their tourist vehicles use a network of unsealed sand (or mud in the rainy season) tracks [see McNutt (1996) for further details].

## 3 Data

### 3.1 Collar Data

Between 2011 and 2022, 26 free‐ranging wild dogs from 19 packs were fitted with GPS collars programmed to collect fixes either based on wild dog activity (Wilson et al. 2013; Hubel et al. 2016) or every 3 h (VERTEX PLUS, Vectronics Aerospace). Individual dogs were darted from a vehicle using a combination of xylazine (55 mg), ketamine (50 mg), and atropine (1.1–1.2 mg) in order to fit GPS collars and were reversed after an hour with yohimbine (4 mg) or atipamezole (5.5 mg); immobilization procedures followed standard protocol established by McNutt (1996). Collars were preferentially fitted to resident dominant/breeding individuals, allowing us to avoid potential dispersal forays and have confidence that individual movement data reflected pack movement (Jordan et al. 2014). We used a combination of quality indicators measured by the activity‐based collars and established procedures (Urbano and Cagnacci 2014) to clean and sort GPS data into a single trajectory per pack by combining collar data across individuals of a pack. Data were regularized to a 3‐h resolution using the workflow from the R package “amt” (Signer et al. 2019) for continued processing.

### 3.2 Demographic, Social, and Behavioral Observations

Botswana Predator Conservation (BPC) has been monitoring the subpopulation of African wild dogs in the study area since 1989. BPC's long‐term, exhaustive database of observed wild dogs in their study area identifies individuals by their unique pelage patterns. Each sighting in the study area is geo‐tagged, timestamped, and associated with pack‐specific information including pack size, breeding status, and composition (e.g., a list of all individual wild dogs present). These observations allow us to determine time‐since‐pack‐formation, a proxy for pack experience (e.g., the time between a given observation and the first time the pack, specifically the male and female dominant pairing, was observed), pack size (e.g., the number of adults over the age of 1 year present in the pack) and pup presence (e.g., pups present or not) for each recorded sighting. The database of individual wild dog detections is also used to facilitate the collection of individual‐ and pack‐specific behavioral observations, including scent marking behavior, hunting behavior, mating behavior, and movement state (e.g., resting, moving, coursing; see Table S2b). Wild dog packs are followed in the field (on and off road) and individual behaviors are observed using critical incident sampling (Altmann 1974). Behavioral observations are recorded using a custom form on the Kobo Collect app on an Android device (https://www.kobotoolbox.org/). Observations of individuals and packs are made in vehicles between 10 and 200 m away, depending on whether dogs are resting or moving. All behaviors are recorded directly onto the Android device according to behavioral protocols established by Jordan et al. (2013).

First we categorized behavioral observations of a single, pack‐specific entity into “follows.” A follow began at the time and place the observer first saw the pack, and ended at the time and location that the observer left (or was left by) the pack. This protocol resulted in a series of follows comprised of GPS locations and timestamps, each associated with specific behaviors and individuals who enacted the behavior. Importantly, critical incident sampling implies that a location was only recorded when the behavior occurred; by measuring occurrence, we could infer when the behavioral state changed from the previous state. Follows were then interpolated evenly over time for continuous, observational tracks. See S2 for more details.

Our cumulative behavioral observations resulted in two sets of GPS locations: the first was an accumulation of all observed scent marking locations, with each location associated with a pack, and a timestamp (hereafter referred to as “SM data”). These were collected between 2010 and 2022. The second was a dataset of pack‐specific follows, collected between 2017 and 2022, where each location represents a distinct behavior associated with a pack, a follow ID, the pack's social information, and a timestamp (hereafter referred to as “follow data”).

### 3.3 Environmental and Territorial Covariates

To test the relative influences of habitat composition and territorial layouts of conspecifics on scent marking behavior, we spatially joined all points in our datasets to several distance to land cover raster layers. Land cover data included roads, human settlements, bodies of water (seasonal pans), four vegetation types—grassland, floodplain, mixed woodland, and mopane woodland (Bennitt et al. 2014), and covered habitat (derived from a combination of the mixed woodland and mopane woodland vegetation types to isolate habitat types with increased cover). Additionally, all GPS locations and follow data were assigned seasonal classification information, designating whether the observed scent marking location or behavioral observation occurred during the early flood season (April–July), late flood season (August–November), or rainy season (December–March) (Bennitt et al. 2014). These seasonal classifications are especially relevant to the use of seasonal pans, which have varying amounts of water based on the season and their depth (begin filling in the rainy season, full in the early flood season, and dried out in the late flood season). Each used and available scent marking location was also associated with spatiotemporal territorial data from neighboring conspecifics. Territorial data represented neighboring and own space use (kernel density estimates per Signer and Fieberg 2021) from the past 7, 14, or 30 days. See S4 for more details. All environmental and territorial covariates were standardized (mean‐centered and scaled by standard deviation) and tested for multicollinearity using Pearson's correlations. Only terms with a correlation value of |*r*| < 0.6 were included in the same models (Hinkle et al. 2003).

While we prepared point‐specific covariate data (e.g., distance of roads between used/available points) for observed scent marking locations, in our final analysis we aggregated the environmental and territorial data over the entire follow. Specifically, we isolated the values (e.g., distance to road, distance to seasonal pans, neighbor's territory UD) at the first and last points of the follow to generate two types of covariates: the first was a difference in value, as defined by the covariate data at the first point of the follow minus the same covariate data at the last point. This covariate had a continuous distribution. The second was a simplified version of the difference in value, where, if the difference between the two points was negative, then the covariate equaled 0, and if it was positive, the covariate equaled 1. These aggregate‐level covariates were also mean‐scaled and tested for multicollinearity using Pearson's correlations.

## 4 Model Selection

We ran three analyses to investigate the environmental, territorial, and social drivers of scent marking behavior in African wild dogs. Our principal questions were as follows: (A) whether the relative location of neighboring packs was more important than, or mediated the strength of, a given habitat type in dictating scent mark placement, (B) whether and how neighboring packs altered behavioral decision‐making (e.g., scent marking versus engaging in other behaviors) at a fine scale, and (C) whether and how the social structure of packs, specifically pack experience, pack size, and pup presence, mediated scent mark placement and behavioral decision‐making (e.g., scent marking versus hunting) in areas with increasing neighbor presence. All model fitting and subsequent investigation were performed in R (R Core Team 2023).

We performed three model selection and averaging procedures to investigate these questions. First we modeled the relative probability of wild dog scent marking locations (*SM data*) across their territories based on environmental features and their neighbors' locations (analysis 1). Next we utilized our *follow* data to model the probability of scent marking in response to neighbors, habitat, and the social composition of the focal pack (analysis 2). We used findings from both our first two analyses to consider how differences in order of selection influences territorial behaviors (Johnson 1980). Lastly, using the same predictor variables, we modeled how packs balance territorial maintenance (e.g., scent marking observations) versus territorial use (e.g., hunting observations) by aggregating social and environmental data along each follow (*aggregated follow* data; hereafter referred to as *AF* data).

### 4.1 Relative Scent Marking Probability Within a Pack's Territory

Our first goal was to determine how territorial layout and habitat affects where wild dog packs scent mark. The locations of all observed scent marking behaviors were extracted from an 11‐year period from 2010 to 2021 to generate our *SM* data. We used GPS collar data from the 90 days prior to each observed scent mark to create territories from 95% utilization distributions that were seasonally restricted, and so comprised relevant available points for any given observed behavior (both in terms of environmental and seasonal changes, but also shifting pack dynamics) (O'Neill et al. 2020; Hansen et al. 2024). We randomly selected 15 available scent marking locations per observed scent marking event within each pack's temporally‐restricted territory (Fieberg et al. 2021). In total, we analyzed 2051 scent marking locations from 18 wild dog packs, with a mean of 114 observations per pack. All observed and available scent marking locations were spatially joined with environmental and territorial covariate data. We ran a resource selection function (RSF; Manly et al. 2002) using the workflow from the R package “amt” (Signer et al. 2019) to compare the environmental and territorial covariates between used (y=1) and available (y=0) locations. Assuming there are j=1,…,J packs, each pack will have $i=1,\ldots ,I_{j}$ locations, both used and available, per pack j. Using an exponential regression characteristic of the RSF, which is standard for Bernoulli distributed data, we modeled the relative probability (relPr) that a pack scent marks at point $y_{\mathrm{ij}}$ with a covariate vector $x_{1n}$ such that

(1) $$\text{relPr}\left(y_{\mathrm{ij}}=1|x_{1n}\right)=\exp\left(\alpha_{j}+x_{1}\beta_{1}+\ldots +x_{n}\beta_{n}\right)$$

where $\alpha_{j}$ is our pack‐specific intercept (meant to account for pack‐level differences in numbers of observations), $x_{1}\ldots x_{n}$ is our vector of relevant habitat and or territorial covariates, and $\beta_{1}\ldots \beta_{n}$ is the vector of affiliated estimated coefficient per covariate. We included weights in our maximum likelihood estimation process, per Manley et al., based on whether a given point was a known scent marking location (W=1) or an available location (W=100) (Manly et al. 2002). We executed a model selection procedure where we identified a biologically relevant candidate set of models based on specific criteria: (1) the model must include linear terms for distance to roads, grassland, seasonal pans, and permanent water, which were all found to be important for wild dog habitat selection and latrine in previous literature (e.g., see Hansen et al. 2024; Claase et al. 2022; Abrahms et al. 2016), and (2) only contain interaction terms with either residency or neighboring pack territory, but not both, in order to avoid model overfitting. We tested this model set with a limited set of biologically relevant habitat, territorial, and social linear terms (see Table S1 for details), and a specific set of interaction terms between territorial covariates and either habitat or sociality (Table S1) to test our predictions (Table 1). Specifically, we tested interactions between territorial covariates and (1) roads, (2) seasonal pans, and (3) number of co‐occurring neighbors given that wild dogs use latrines that tend to be closer to roads, pans, and occur in boundary areas between neighbors, where there are more co‐occurring packs (Claase et al. 2022). We also included a three‐way interaction between flooding season, seasonal pans, and a territorial term to account for differing water levels at seasonal pans across the study's range (Table S1). Lastly, we also included a null model (random intercept only), and used model selection to find the best performing model and verify our top model performed better than the null. We averaged the top models within 2 AICc to make inferences on what factors influence scent marking behavior in wild dog packs (Burnham and Anderson 2004), and compare the relative importance of habitat versus conspecific presence. Specifically, we averaged models by multiplying each covariate by the model's AICc weight, and summing across all models, to infer model average covariates (Anderson and Burnham 2002). To interpret our model findings and test our predictions, we calculated the relative selection strength (Avgar et al. 2017) of terms in the averaged model which were relevant to our predictions.

**TABLE 1:** A list of predictions with an associated number, and the analysis in which we tested the prediction. A detailed description of each analysis begins below.

Prediction	Number	Data used	Analysis tested
The relative locations of conspecifics should have a greater influence on scent mark placement than habitat	P1	*SM follow*	1 2
Packs should scent mark in open, preferred habitat (e.g., roads, seasonal bodies of water) in their own territories to maximize advertisement potential of scent to intruders	P2	*SM follow*	1 2
Packs should scent mark more in heavily contested areas (e.g., more neighbors) than when intruding a neighbor's territory (e.g., fewer neighbors)	P3	*SM follow*	1 2
Packs should mediate their decisions to advertise their presence relative to other behaviors based on their social composition	P4	*follow*	2
Pack social composition, specifically pup presence, experience, and size, should mediate time spent utilizing versus patrolling territories	P5	*AF*	3

### 4.2 Probability of Scent Marking Versus Other Behaviors Along a Follow

Unlike the previous analysis, the structure of our interpolated *follow* data allowed us to model the probability of scent marking during observed, behavioral sessions against known, non‐scent marking locations. In our previous analysis we compare known scent marking locations against other available scent marking locations within pack territories, which may confound locations where packs are likely to be with where packs are likely to scent mark. In this analysis, however, we take into account where packs are and specifically investigate the true probability of scent marking. We analyzed data from 108 follows over 10 packs (mean of 10.8 [±7.33] follows per pack). Our dataset contained 536 observed scent marking locations over all follow data (mean of 59.6 [±43.6] scent marking locations per pack). We utilized all observed scent marking locations along follows as our “used” points (y=1). All other behavioral locations along follows (e.g., where animals engaged in other behaviors) were our “unused” points (y=0). Utilizing a logistic regression, we then modeled the probability pack j scent marks along follow f at point i such as

(2) $$P\left(y_{\mathrm{ijf}}=1\;|\;x_{1n}\right)=\frac{\exp\left(\alpha_{\mathrm{jif}}+x_{1}\beta_{1}+\ldots +x_{n}\beta_{n}\right)}{1+\exp\left(\alpha_{\mathrm{jif}}+x_{1}\beta_{1}+\ldots +x_{n}\beta_{n}\right)}$$

where $\alpha_{\mathrm{jif}}$ is a nested intercept, specific to each pack‐follow combination, and our covariates and associated coefficients are the same as in Equation (1). Given that we included a nested intercept in our model (follow per pack) to account for autocorrelation within a given follow and the amount of follow data we had per pack, we removed all follows from the dataset which contained no scent marking observations. The results from this model can be interpreted like a resource selection probability function (RPSF; Lele and Keim 2006), because our probability of scent marking is known relative to unused scent marking locations. In this RPSF model set we tested two additional terms, which were interactions between neighboring territory and (1) pack size and (2) pup presence, while removing seasonal interaction terms (e.g., season pans ~ flood) since our more limited dataset precluded coarser‐scale, seasonal interactions and three‐way interactions. We used the same filtering criteria to limit our candidate model set to those models with biologically relevant information, and replicated our model selection and averaging protocol as above. We determined which factors were most important in predicting probability of scent marking behavior, specifically considering the importance of conspecific presence relative to habitat type, any interactions between territorial terms and environment, and our social interaction terms (Table 1). We calculated the predicted probability of scent marking from relevant covariates, and calculated the area under the curve to evaluate overall model performance (James et al. 2021).

### 4.3 Behavioral Trade‐Off Between Marking and Hunting Along a Follow

To consider whether and how packs managed territorial maintenance (i.e., where they chose to scent mark vs. where they chose to hunt), we directly compared how habitat, territorial layout, and pack composition influenced territorial maintenance (e.g., scent marking) relative to territorial use (e.g., hunting). We isolated 123 follows over 9 packs for our *AF* dataset. Follows lasted 135.27 min and covered 1.78 km on average. For each follow, we calculated two standardized ratios of behavioral‐specific events per hour per follow, specifically scent marking‐per‐hour to hunting‐per‐hour (see S3 for more details). We divided the markings‐per‐hour by the hunting‐per‐hour to get a ratio of scent marking to hunting behaviors for each aggregated follow. Lastly, we took the log of this ratio so our data followed a Gaussian distribution. Following convention for normally distributed data, we modeled the log‐ratio, y, per pack j and follow i, such as:

(3) $$y_{\mathrm{ij}}=\alpha_{j}+x_{1}\beta_{1}+\ldots +x_{n}\beta_{n}+\varepsilon$$

where our intercept is the same as Equation (1).

Model selection was used to determine how habitat, territorial layout, and sociality influence scent marking behavior relative to hunting behavior (Table 1). Given the size of the dataset and our interest, we had a limited candidate set of models specifically testing whether relative direction to roads along a follow, relative exposure to neighbors, exposure to vegetation cover, and pack composition attributes (pack size, pack experience, and number of overlapping neighbors) predicted the marking to hunting ratio during a follow. We assessed model fit using a *k*‐fold cross‐validation procedure with 10 folds, calculating the average root mean squared deviation (RMSE) across all folds. We then compared the mean RMSE to an RMSE of a baseline prediction (e.g., the difference between observed data and the mean of all observed data). RMSE in particular is commonly used to evaluate the predictive capacity and/or accuracy of linear regression models (James et al. 2021). We then used a paired t‐test to compare RMSE values from the averaged model against RMSE values from a baseline prediction within each fold to determine whether the difference in RMSE was statistically significant.

## 5 Results

### 5.1 Relative Scent Marking Probability Within the Territory

**FIGURE 1:**
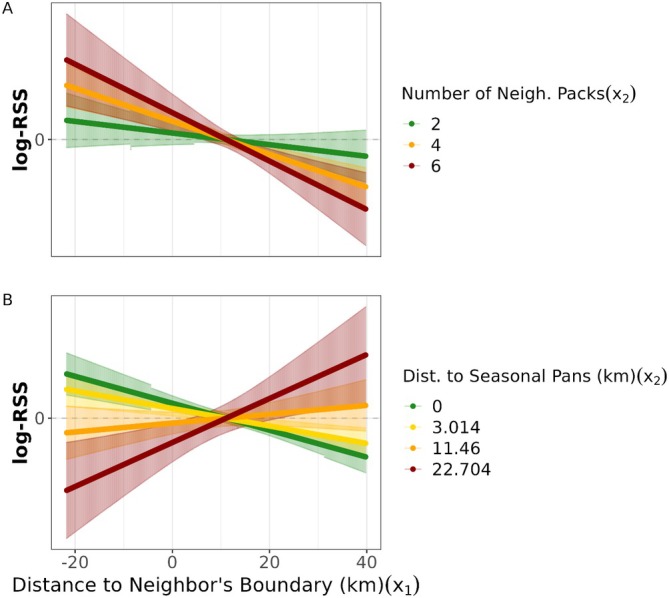
The log‐relative selection strength (log‐RSS) of wild dog pack scent marking behavior is shown across all possible distances from a neighbor's 14‐day territory boundary (where smaller values indicate greater neighboring use, and larger ones indicate lesser neighbor presence). We show how relative selection strength for scent marking locations changes across a neighbor's territory based on two different values of *x*_2_: (A) the number of co‐occurring neighbors, and (B) the distance to seasonal pans. We used the minimum (close), mean (medium), 3rd quantile (farther), and maximum (far) distances from both roads and pans across the dataset to determine distance‐specific responses to neighboring boundaries. Packs show mediated scent marking behavior within neighbors' territory based on habitat features and the number of overlapping neighboring packs.

Three models were within 2 ΔAICc of the minimal model and subjected to model averaging. All top models outperformed the null model. Packs showed strong selection for scent marking outside of their territory's core (e.g., 14‐day core; β=1.04±0.03). They selected for scent marking locations in neighboring territory boundaries overall (14‐day boundary; β=−0.45), and favored doing so when near seasonal pans (Figure 1B). Additionally, seasonality altered the relative probability of scent marking near seasonal pans (Figure S5). The relative probability of scent marking increased when a given location within a neighbor's boundary had more overlapping neighbors (β=−0.23±0.06; Figure 1A). In terms of habitat, packs avoided scent marking near grassland (β=0.033±0.043), while selecting for roads (β=−1.05±0.072) and pans (β=−1.12±0.092). They also had negligible selection for scent marking on rougher, more elevated terrain (β=−0.001±0.02) and near permanent water (β=−0.001±0.02).

### 5.2 Probability of Scent Marking Versus Other Behaviors Along a Follow

**FIGURE 2:**
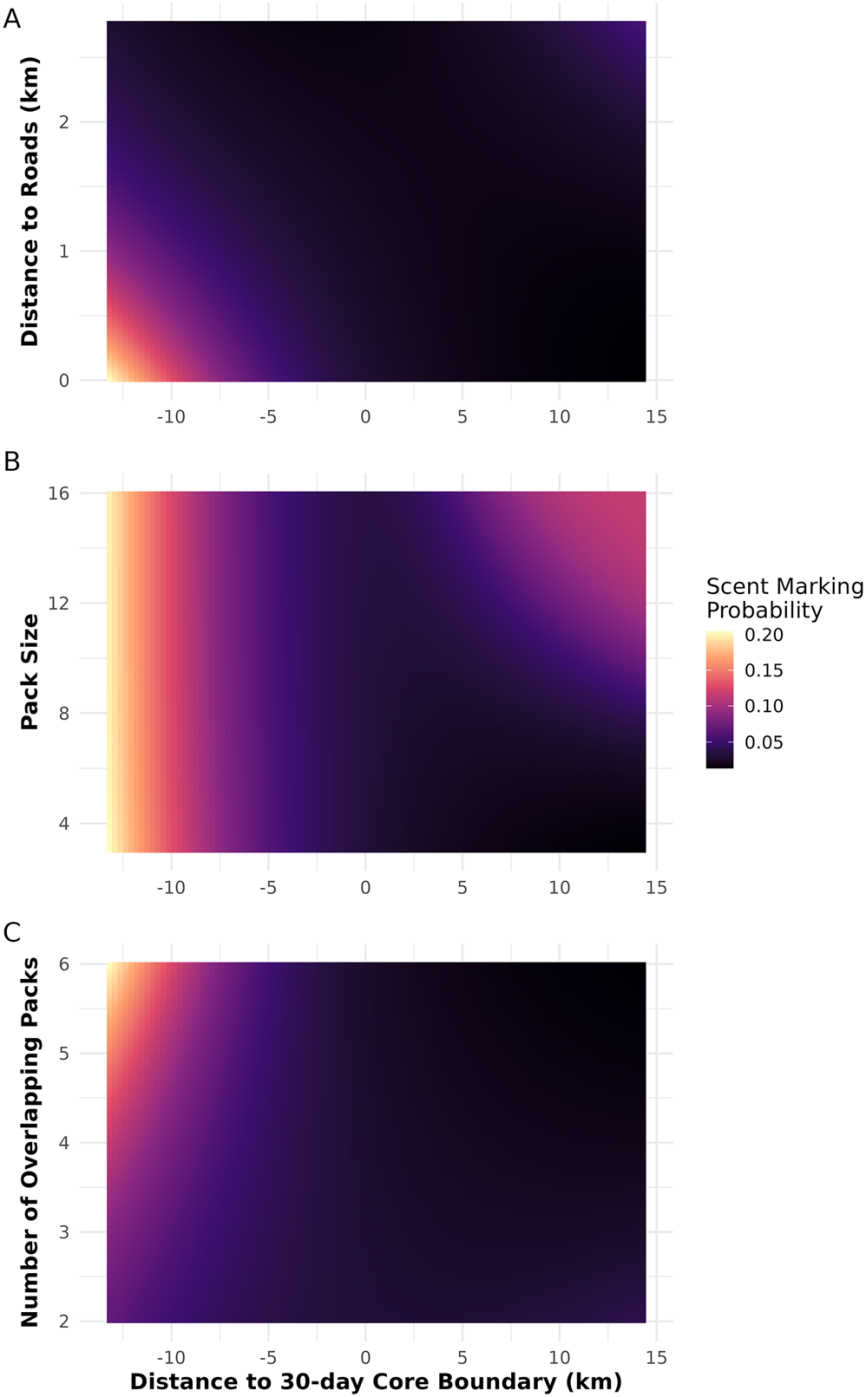
Contour plots depicting how the probability of scent marking changes relative to a pack's own core territory boundary, where negative distances are areas within the core and positive distances are outside the core. We specifically highlight how scent marking probability changes across a pack's territory based on (A) distance to roads, (B) focal pack size, and (C) the number of neighboring packs that also occur in the area. We calculated the probability of scent marking along all possible distances from roads across the dataset along all possible distances from the pack's 30‐day, core territory boundary. Similarly, we determined the probability of scent marking at each discrete number of neighbors and all possible pack sizes.

Our model selection resulted in 7 models within Δ2 AICc, from which we derived model averaged estimates and effect sizes. The area under the curve value of our averaged model was 0.739, indicating that the model has a moderate discrimination ability in predicting scent marking probability. Packs were more likely to scent mark than engage in other behaviors in their neighbors' territory (β=0.19±0.2) along a follow. However, territorial layout mediated the probability of scent marking in certain habitat types. Specifically, when in their own territory (30‐day distance to core) packs preferred marking closer to roads (Figure 2C). The number of neighboring packs and pack size both mediated the probability of scent marking: while all packs were likely to scent mark within their core territories, larger and medium‐sized packs were more likely to scent mark outside of their core territory (Figure 2B), yet all packs were more likely to scent mark within their core territory when the area was increasingly contested (Figure 2A). Packs without pups (representing less than 10% of all observed behaviors) had a higher probability of scent marking within their core territory than packs with pups (Figure S6), although these packs were also much more likely to scent mark in general (β=0.04±0.16) suggesting seasonal differences in territory use may be driving this pattern. In terms of habitat features, packs preferentially marked near roads (β=−0.26±0.15) and pans (β=−0.32±0.13), with a slight preference for grasslands as well (β=−0.06±0.10). They also lightly avoided water (β=−0.0.04±0.10) (Figure S6).

### 5.3 Behavioral Trade‐Off Strategy

We identified 12 models within 2 ΔAICc of the minimum which were averaged into one model. We took the RMSE from our averaged model in a 10‐fold cross‐validation procedure, and found the averaged RMSE across all 10 folds (mean RMSE = 6.712) slightly outperformed the RMSE from a naïve model of averaged predictions (mean = 6.897). Despite the small magnitude of difference, a paired *t*‐test comparing the RMSE values of the averaged model to that of the baseline across all 10 folds suggests our averaged model is performing significantly better than the baseline (*p* = 5.800e^−11^). We found that pack size, pack experience, the average distance to roads and to seasonal pans over the follow, and whether the pack was entering or leaving a neighbor's territory, were the most important factors in determining behavioral trade‐off strategies in wild dog packs. Overall, as packs entered neighboring territories over the course of the follow, they were more likely to scent mark than hunt (β=0.936±0.607). When packs were, on average, closer to pans over the course of the follow they were more likely to scent mark (β=−0.935±0.677). Mean distance to roads, however, had the opposite effect (β=0.677±0.724).

**FIGURE 3:**
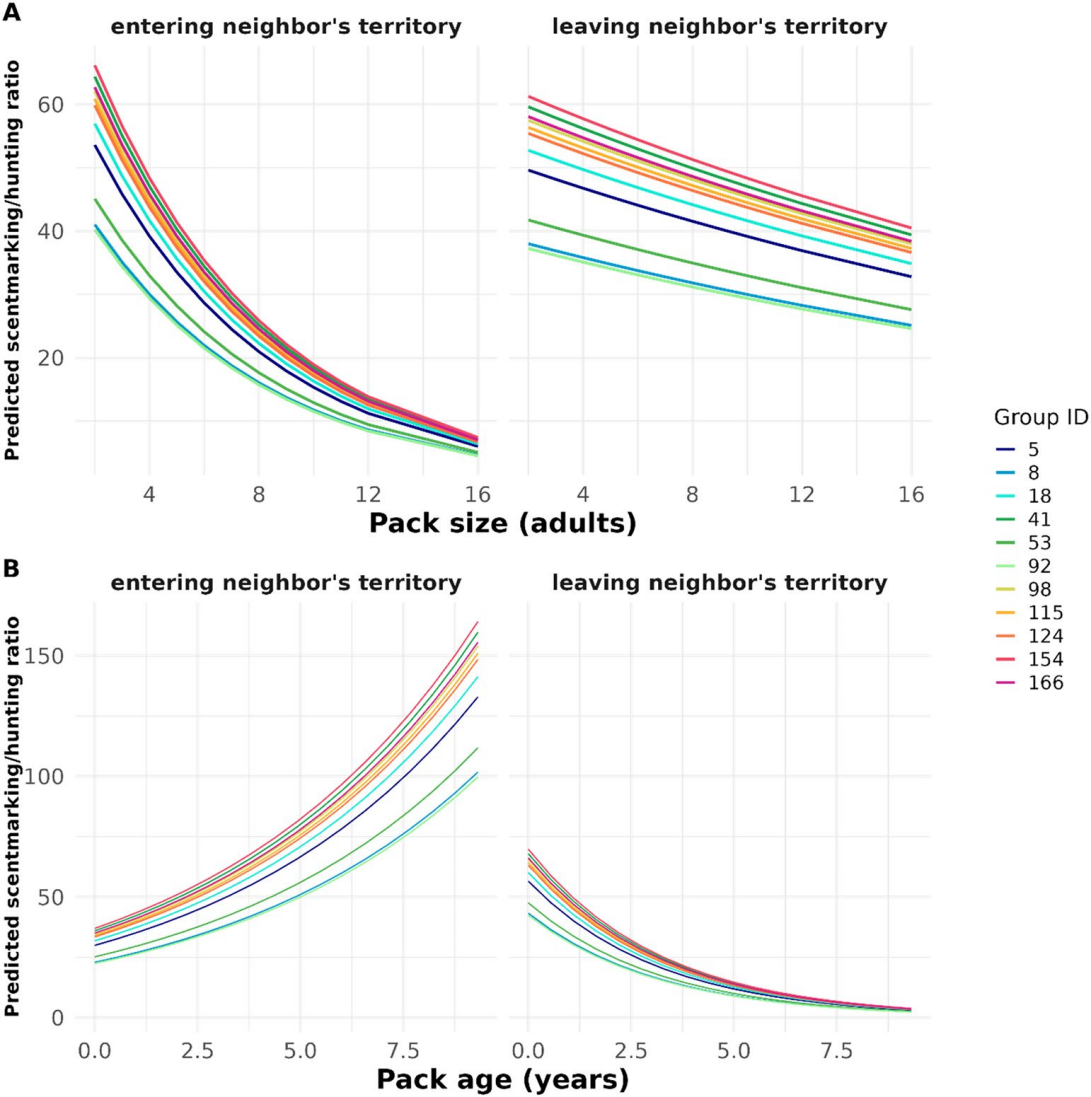
Here we show predicted scent marking to hunting ratios for each individual pack, based on whether they are entering or leaving a neighbor's territory. An increase in the *y* axis translates to increased marking behavior relative to hunting behavior, suggesting that packs are actively patrolling and/or concerned with leaving signs of presence. A smaller marking behavior to hunting ratio could indicate both increased use of a given area and/or cryptic behavior, where a pack either does not want to, or is not concerned with, leaving signs of presence. Given that our model had a random intercept on pack, we show here the predicted ratios for each pack‐intercept. (A) shows the change in behavioral ratio across different pack sizes, while (B) shows the change in ratio across pack ages in years.

Pack size and pack age both affected behavioral strategies in wild dog packs. Larger packs were more likely to hunt than scent mark than were smaller packs (β=−0.074±0.275), while age, on its own, had a very small negative effect on the marking to hunting ratio (β=−0.003±0.002). However, each of these social attributes also interacted with neighboring territory to influence how packs alternated between hunting and scent marking when in neighboring territory. While more experienced packs increased their marking behavior when the follow entered a neighbor's territory relative to younger ones, larger packs were much more likely to hunt in neighbors' territories than smaller packs (Figure 3). When the follow was leaving a neighbor's territory, the relative difference in marking to hunting across pack size and pack age was much less variable across ages, suggesting a larger response to increased risk of encountering a neighboring pack (Figure 3).

## 6 Discussion

We ran three sets of analyses to investigate the environmental, territorial, and social drivers of scent marking behavior in African wild dogs. First, we determined how wild dogs scent marked across their territories in relation to habitat features and their neighbors. Next, we utilized a unique dataset of wild dog follows to understand the probability of scent marking based on the territorial layout of conspecifics, environmental features on the landscape, and social composition of focal packs. Lastly, we aggregated the ratio of marking to hunting events per follow to untangle how packs balance territorial maintenance versus territorial use (e.g., food acquisition) based on their location relative to neighbors, habitat features, and the social characteristics of the pack itself. Critically, all three analyses revealed the importance of neighboring conspecifics in dictating the probability of scent marking at a given location, and how territorial layout interacts with habitat features and social characteristics to alter scent marking strategies in wild dog packs across spatial scales. While previous theoretical work has demonstrated the importance of individual differences in managing conflict outcomes in territorial simulations, this study expands upon our understanding of the decision‐making of territorial animals in a social context using empirical data (Hamilton and McNutt 1997). Combined, these analyses place scent marking within the context of habitat selection and space use of African wild dogs and exemplify the importance of contextualizing movement and behavior in the social dynamics of the landscape.

Across all three analyses, there was a clear effect of sociality on mediating behavior in neighboring territories. In the first two analyses, the number of co‐occurring neighbors altered the relative or true probability of scent marking. In our second analysis, both pup presence and pack size were additionally important in mediating the probability of scent marking. Most notably, larger packs were more likely to scent mark than engage in other behaviors when outside of their core areas. In our third analysis, the size and experience of the focal pack directly influenced the probability of scent marking as compared to hunting behaviors in neighboring territory. In tandem, these results suggest that packs are aware of not only who their neighbors are and how many there are, but also how their own social composition may relate to their competitors (e.g., smaller or less experienced). Theory suggests that the evolution of sociality relies on individual recognition, and, in lizards, the ability to recognize individual scent has been shown to mediate their scent marking behavior (Carazo et al. 2008). On a larger scale, other species, specifically long‐tailed tits ( *Aegithalos caudatus* ) and meerkats (*Suricatta suricatta*) are known to mediate their response to conspecifics based on relative social composition, such as relatedness and group size (Ellison et al. 2020; Bateman et al. 2014). However, it has been less clear how social composition affects behavioral decision‐making on a fine scale. While we know that scent marking patterns across a territory extend to energetic use at the fine scale (Young and Monfort 2009), this effect has not been explored in a social context. Our paper illustrates how behavioral decisions can be mediated at the socio‐spatial interface and have impacts extending to territorial layout and foraging decisions (Webber et al. 2022).

The apparent differences in packs choosing when to scent mark versus hunt indicate that packs utilize a behavioral trade‐off strategy that relies on both spatial‐social organization and social context. Territorial overlap among co‐occurring individuals is common throughout many species besides wild dogs, and, in certain contexts, invasions into neighboring territories are beneficial (Potts et al. 2019). Despite this knowledge, the majority of literature considering invasion strategies for territorial animals is based on simulation models and not behavioral observation (but see Potts et al. 2019). Even when territories are defended, simulation models show that intrusions pay off for a given individual/group (Hinsch and Komdeur 2010). Extra‐territorial forays or extensions can emerge in response to limited resource availability (Potts et al. 2019), to scout alternate potential territorial space (Kamler et al. 2019), or for additional mating opportunities (Deuel et al. 2017). It is less clear, however, when and how individuals or groups should manage scent marking decisions while using areas with increasing neighbor presence. In giant sea otters ( *Pteronura brasiliensis* ), individuals utilize a combination of marking and vocalizations to establish dominance when entering a neighbors' territory to contest space (Potts et al. 2019). However, not all forays necessitate advertisement if there is not an explicit purpose to extend territorial area (Mancinelli and Ciucci 2018). In our system, the behavioral decision‐making involved in managing movement in neighboring territory (e.g., when packs hunt in neighbors' territories and whether they advertise their presence) and movements in their own territorial areas which are contested is likely important in mediating the risks of potentially deadly interspecific conflict (Jordan et al. 2017). Older wild dog packs are more likely to scent mark when entering a neighbor's territory, indicating their experience on the landscape may affect their desire to advertise their presence to neighbors. Given their knowledge of the area, experienced packs may have stronger preference for existing territorial space (Merkle et al. 2017), which may be reinforced by the memory of previous interactions with neighbors or neighbors' scent (Potts and Lewis 2016). However, larger packs, likely also a formidable competitor relative to smaller packs, are less likely to scent mark than to hunt when entering their neighbor's territory. Given that larger packs have more mouths to feed, pack size may be a better indicator of foraging needs in a pack.

In our system, larger packs may also be more experienced, even if this is not always the case. While an interaction between age and experience did not significantly improve model fit in our procedure, our limited dataset may preclude the importance of this additional social interaction on pack behavior. For instance, larger and more experienced packs may prioritize scent marking in other neighbors' territories which are rival competitors (e.g., other large and experienced packs) but prioritize hunting when in the neighboring territory of smaller, less experienced packs which do not constitute a threat. Indeed, other work in our study area has found that larger packs are more likely to overmark other large packs than small packs, and small packs are unlikely to overmark larger packs (Claase et al. 2024). In lizards, for instance, males increase scent marking behavior in response to the scent from rival males which constitute a larger threat (Carazo et al. 2008). Similarly to wild dogs, chimpanzees can engage in deadly intra‐group aggression, specifically during their trade‐mark territorial patrols (Wrangham and Glowacki 2012). When tested against a range of ecological and social predictors, the single best predictor of patrolling behavior in chimpanzees was group size, which indicates that social dynamics are hugely influential in the territorial decision‐making of species which risk deadly inter‐group encounters (Mitani and Watts 2005).

Between our RSF and RPSF, we found relatively similar patterns of selection and avoidance across conspecific territories and habitat types with regard to scent marking behavior. Principally, packs preferred scent marking near roads and pans, and increased marking behavior with more overlapping neighbors. These results are consistent with the idea that packs may balance managing cryptic invasions with claiming contested areas that are known as heavily overlapping. For instance, in banded mongooses ( *Mungos mungo* ) and white‐faced capuchins ( *Cebus capucinus* ) encounters that occur closer to territorial cores are more aggressive than boundary areas, where there is likely more overlap and, especially in wild dogs, a decreased chance of aggressive, lethal encounters (Furrer et al. 2011; Crofoot et al. 2008). Overall, the RSF results mirror other findings in our study area, which show a general avoidance of floodplains and selection for roads when it comes to the locations of African wild dog latrine sites (Claase et al. 2022). Importantly, both analyses demonstrate that territoriality mediates habitat selection in wild dog packs (Hansen et al. 2024), and, by using behaviorally specific habitat selection, reinforces the idea that the needs of territorial maintenance (e.g., scent marking) may be a principal driver in mediating habitat selection in response to conspecifics.

While we were surprised in our second analysis to find that packs avoided scent marking along roads when in neighbors' territory, this finding was supported in our final analysis, where packs were more likely to mark when the average distance of the follow was away from roads. Our second analysis suggests that perhaps roads are utilized in neighboring territories to facilitate quick and efficient movement, and so perhaps packs are more likely to move (among other behaviors) than scent mark along roads in neighboring territories (Abrahms et al. 2017). Quick movement may also facilitate better hunting behavior, as it allows packs to travel more ground to discover more prey options, also explaining our third analysis result. However, given that packs select for scent marking near roads in their own territories, this behavior suggests that roads are an important mode of scent advertisement. Further work is required to consider the interplay between selection for scent marking near roads when the focal pack is large versus small. As seen in Figure 2B, large packs are more likely not only to scent mark in neighboring territories, but additionally, as suggested by Claase et al., may favor scent marking when in neighboring territory of other large packs (e.g., viable competitors) (Claase et al. 2024). Additionally, smaller packs are less likely to overmark large packs at latrines, and therefore could also be less likely to overmark them along roads where latrines tend to be located (Claase et al. 2024, 2022). Given that the average and maximum pack sizes in our dataset are 6.266 and 16, respectively, but there have been observed pack sizes in our study area of up to 30, our dataset may not allow for a full understanding of how relative social composition (e.g., difference in pack size) interacts with both neighbor's location and habitat type.

Future research should continue to not only contextualize habitat selection, movement, and behavior in sociality, but also consider how fine scale behavioral choices lead to larger scale movement mechanisms. While our study system has the environmental and social contexts to facilitate collection of detailed behavioral observations, the improvement of both biologging technologies and resolution are facilitating greater behavioral observations vis‐à‐vis remote sensing (Burke et al. 2019; Duporge et al. 2021; Nathan et al. 2022). By investigating behavioral decisions along the socio‐spatial interface, our study contributes to the compounding evidence that conspecifics are strongly influential on wildlife movement decisions across scales (Avgar et al. 2020; Smith et al. 2022).

## Author Contributions

**K. Whitney Hansen:** conceptualization (lead), data curation (supporting), formal analysis (lead), funding acquisition (supporting), investigation (lead), methodology (lead), project administration (equal), validation (lead), visualization (lead), writing – original draft (lead), writing – review and editing (lead). **Neil R. Jordan:** conceptualization (supporting), data curation (lead), supervision (lead), writing – review and editing (supporting). **Megan J. Claase:** conceptualization (supporting), data curation (lead), writing – original draft (supporting), writing – review and editing (supporting). **J. W. McNutt:** conceptualization (supporting), data curation (supporting), funding acquisition (lead), supervision (supporting), writing – review and editing (supporting). **Alan Wilson:** data curation (supporting), funding acquisition (supporting), writing – review and editing (supporting). **Christopher C. Wilmers:** formal analysis (supporting), investigation (supporting), methodology (supporting), supervision (lead), writing – review and editing (supporting).

## Conflicts of Interest

The authors declare no conflicts of interest.

## Supporting information


Data S1.

## Data Availability

Given that our study species is endangered and the locational data can be used to exacerbate conflict in our study area, data can be made available to specific individuals upon reasonable request by contacting J.W.M., N.R.J., and M.J.C.
