# Supplementary material for: To Hunt or Patrol? Social Composition and Location Mediate Scent Marking Decisions of a Large Carnivore
Source: Ecol Evol. 2025 Jun 18;15(6):e71567. doi: 10.1002/ece3.71567 (PMC12176245; doi:10.1002/ece3.71567)
Supplement: Supplementary file 1 — Data S1. [file ECE3-15-e71567-s001.docx]

Supplemental Materials:

S1: Covariates Tested

Table S1: A summary table of all covariates and interaction terms used in Analyses 1-3, including the covariate name, a description of each covariate, the hypothesis explaining its inclusion in the candidate model set, and a column designating which analysis included the covariate.

| Covariate | Description | Hypothesis | Analysis |
| --- | --- | --- | --- |
| Dist. to seasonal pans: Flood season | An interaction between distance to open pans (seasonal bodies of water) and a categorical covariate designating the time of year (relative to flooding season- early, late, or rainy). | Pans vary in importance seasonally based on available water; packs should vary selection strength for pans based on rainfall/flooding, which dictates whether scent can be found or not. |  |
| Dist. to water | Distance to permanent bodies of water | Wild dog latrines are not located near water, and so packs likely avoid the area giving seasonal flooding that can wash away scent marks. | 1, 2 |
| Dist. to roads | Distance to roads | Packs should mark near roads, which is well-documented canid marking behavior. | 1, 2 |
| Dist. to seasonal pans | Distance to open pans (seasonal bodies of water) | Wild dog latrines are located near pans, and so packs should select for marking near pans | 1, 2 |
| Flood | Season of the year (rainy, early flood, or late flood) |  | 1, 2 |
| Neigh. Terr | A proxy for how intensely a given location is used by neighboring packs; specifically either the maximum UD value of a specific neighbor, the mean UD value across all known neighbors, the distance to the closest UD-outline of a given neighbor, or the average distance to all UD-outlines of all neighbors. All 4 values were calculated from movement data over the past 7, 14, or 30 days, and either for a 50% or 95% UD. | Packs should increase marking probability when entering neighbors’ territory | 1, 2 |
| Neigh. Terr: # of overlapping packs | An interaction between neighbor’s territory and the number of overlapping packs with non-zero UD values at a given location. | In contested areas with more overlapping packs, packs should mark more than when traversing a single pack’s territory | 1, 2 |
| Neigh. Terr: Distance to roads | An interaction between neighbor’s territory and the distance to roads. | Packs prefer marking at roads, and should be more likely to mark at roads in neighbors’ territory, where they also prefer to mark | 1, 2 |
| Neigh. Terr: Distance to seasonal pans | An interaction between neighbor’s territory and the distance to pans. In analysis 1, we included a 3-way interaction between neighbor’s territory, distance to pans, and flood season. | Packs prefer marking at pans, and should be more likely to mark at pans in neighbors’ territory | 1, 2 |
| Own Terr | A proxy for how intensely a given location is used by the focal pack. Specifically either the UD value, or the distance to the UD-outline of the focal pack. UD’s calculated from movement data over the past 7, 14, or 30, and at either 50% or 95%. | Packs should increase scent marking when leaving their own territory | 1, 2 |
| Own Terr: # of overlapping packs | An interaction between neighbor’s territory and the number of overlapping packs with non-zero UD values at a given location. | In contested areas with more overlapping packs, packs should mark more than when traversing a single pack’s territory | 1, 2 |
| Own Terr: Distance to seasonal pans | An interaction between own territory and the distance to pans. In analysis 1, we included a 3-way interaction between own territory, distance to pans, and flood season. | Packs prefer marking at pans, and should be more likely to mark at pans in their own territory | 1, 2 |
| Own. Terr: Distance to roads | An interaction between own territory and the distance to roads. | Packs prefer marking at roads, and should be more likely to mark at roads in their own territory | 1, 2 |
| Neigh. Terr: Pack size | An interaction between neighbor’s territory and the number of adults (> 6 months) in the focal pack. | Larger packs should be less concerned with scent marking in neighbors’ territories relative to smaller packs | 2 |
| Neigh. Terr: Pup presence | An interaction between neighbor’s territory and a categorical covariate signifying whether or not pups are present (0 = not present, 1 = present) | Packs without pups may be dispersers seeking mates, and are less likely to engage in territorial advertisement when in neighboring territories | 2 |
| Cover Difference | The difference in distance to woodland or mopane cover between the first point of a follow and the last point of a follow | Packs should select to mark with increasingly open habitat (and so less cover over the follow) | 3 |
| Neigh. Difference | The difference in neighbor UD between the first point of a follow and the last point of a follow | Packs should increase marking probability when entering neighbors’ territory | 3 |
| Neigh. Simp. | If the neighbor UD value at the first point of a follow is greater than the last point of a follow, then “less”, otherwise “more” | Packs should increase marking probability when entering neighbors’ territory | 3 |
| Neigh. Simp.: Pack experience | An interaction between a simplified version of neighbor UD (e.g. “more” neighbor or “less”) and the number of months since a pack was first observed in the field (a proxy for experience). | Experienced packs will target scent marking in neighbors’ territories relative to younger packs, who may be less aware of boundaries and territorial layout | 3 |
| Neigh. Simp.: Pack size | An interaction between a simplified version of neighbor UD (e.g. “more” neighbor or “less”) and the number of adults (> 6 months) in the focal pack. | Larger packs should be less concerned with scent marking in neighbors’ territories relative to smaller packs | 3 |
| Number of neighbors | The number of known, collared neighboring wild dog packs co-occurring with focal pack | In contested areas with more overlapping packs, packs should mark more then hunt to prioritize advertising presence | 3 |
| Pack experience | The number of months since a pack was first observed in the field, a proxy for experience | More experienced packs will be more efficient scent markers | 3 |
| Pack size | The number of adults (> 1 year old) in a pack | Larger packs are hungrier and will be more concerned with hunting | 3 |
| Pack size: Pack experience | An interaction between the number of adults (> 6 months) in the focal pack and the number of months since a pack was first observed in the field (a proxy for experience). | Older and more experienced packs will prioritize marking and advertising presence consistently to rival packs relative to younger less experienced packs. | 3 |
| Roads Difference | The difference in distance to roads between the first point of a follow and the last point of a follow | Packs should increase scent marking when near roads | 3 |
| Roads simp. | If the distance to roads at the first point of a follow is greater than the last point of a follow, then “away”, otherwise “towards” | Packs should increase scent marking when near roads | 3 |

S2a: Follow Interpolation

To accurately consider locations during the follow which are implicitly associated with a given behavior, follows were interpolated evenly over time (for more details see Claase et al. 2022). This interpolation resulted in GPS tracks where each location, either real or interpolated, was associated with a known behavior, a timestamp, and pack demographic data (e.g. the pack attribute data recorded in any given sighting). We used a small subset of follows where the observer recorded the track continuously to confirm that temporal-interpolation resulted in a more accurate fit to the observed continuous track compared to spatial-interpolation. If the observer could not observe behaviors during a follow due to the pack moving too quickly or being obscured by cover, the session was paused until behavioral observation could resume. Unless a location was marked with the label “pause”, implying the pack was lost by the observer at that location, then we could assume the pack was under observation throughout the follow.

Table S2b: Counts of interpolated behaviors across all follow data.

*
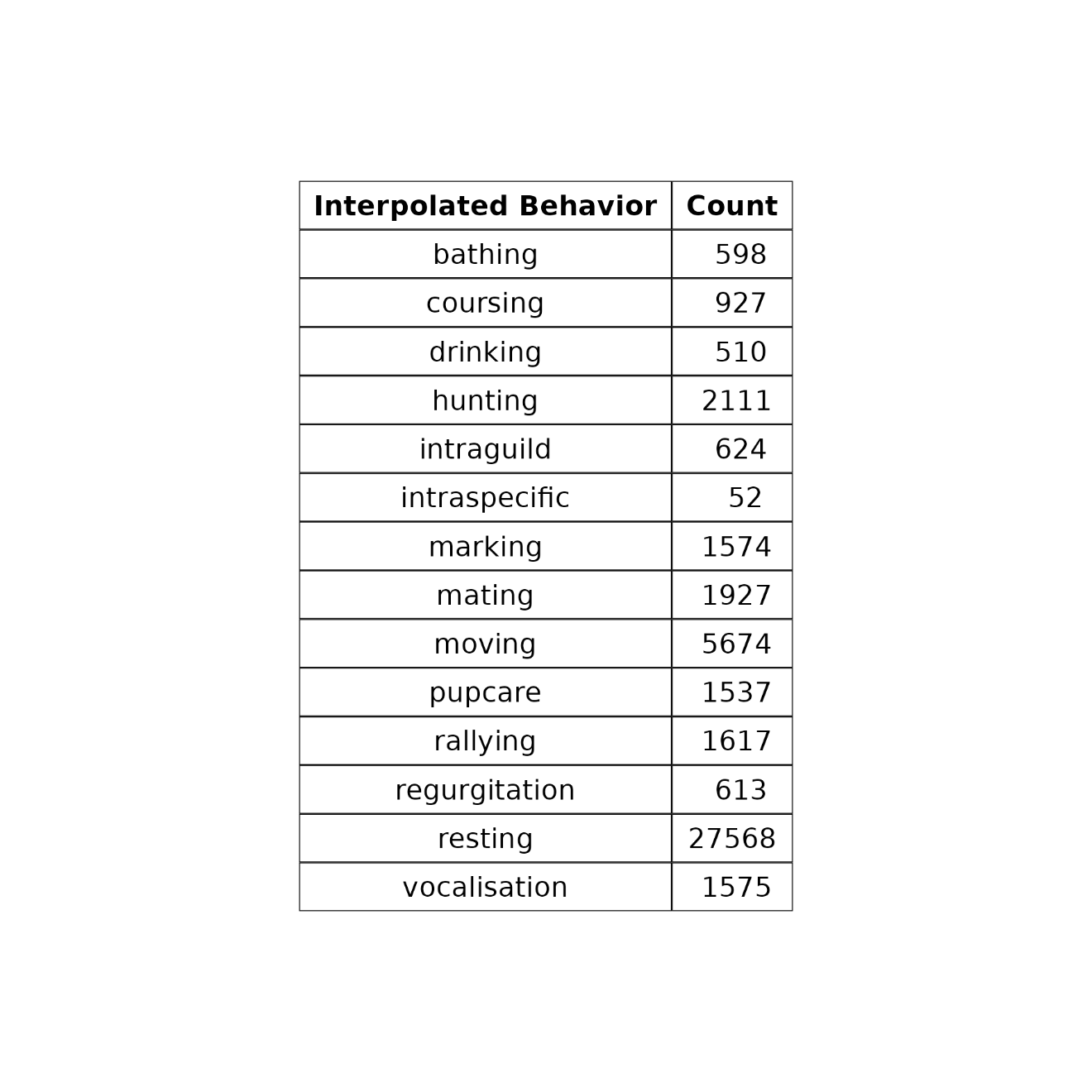
*

S3: Creating the marking-to-hunting ratio

For each follow we removed all behaviors recorded prior to the first “moving” event. This way we only analyzed packs’ movement behavior, which is when they engage in intrapack communication and hunting. We filtered our behavioral dataset to a 5-minute resolution, given that pack members can often engage in bursts of chase events or marking events across individuals, which could artificially inflate behavioral counts and we wanted our analysis to investigate behaviors at a pack-scale. We counted the number of distinct hunting behaviors, which included when dogs were coursing, chasing, and on-kill, and the number of distinct marking behaviors, which included sniffing, overmarking, and marking. We also counted the number of distinct behaviors per follow. We then divided the number of marking behaviors and hunting behaviors by the number of distinct behaviors over the entire follow, which essentially weighted each behavioral count by the overall behavioral diversity along the follow. Lastly, we multiplied each ratio by the total length of the follow in hours to get a behavioral rate per time.

S4: Territorial Covariate Data

For each given day spanning our dataset, we used all collar data to calculate the space use (UDs; Signer and Fieberg 2021) of all wild dog packs at three temporal scales: the past week (7 days), two weeks (14 days), and month (30 days). First we fit movement models to our movement data to account for autocorrelation, before fitting a kernel density estimate per pack per temporal scale (Signer and Fieberg 2021). We extracted the UD value at each point for each temporal scale, for both the 50% and 95% UD. We also extracted each UD’s 50% and 95% isopleth to generate distance-to-UD-outline values (again, for each temporal and spatial scale). For all distance-to-UD calculations, points inside the isopleth boundaries were given a negative value, so that a value of 0 meant the used or control point was on the UD’s outline. For each used and available scent marking location, we had 4 temporal scales of territorial information (for both own and neighboring territories) and 3 spatial scales (the raw UD, distance to 95% UD boundary, and distance to 50% UD boundary). For more details, see Hanen et al. (Hansen et al. 2024). Each dataset was concatenated so that we only included recorded scent marking locations, or entire follows, which had known territorial data for the focal pack and all neighbors for accurate model comparison.

To select the optimal territorial proxy in each of our candidate model sets, we used a Lasso procedure to identify the territorial covariates which best explained variation in relative scent marking probability. We then used the two territorial terms (one for neighbor and one for own territory) with the highest absolute coefficient in our model selection procedure for the subsequent analysis (Zhao and Yu 2006). No two proxies for conspecific space use, or for residency, were included in a single model given that variations in temporal/spatial scale of conspecific space use (or residency) are highly correlated. All point-specific and follow-aggregate covariates were standardized (mean-centered and scaled by standard deviation), and tested for multicollinearity.

For our RSF analysis, our Lasso procedure identified two territorial terms as more responsible for explaining variation in scent marking behavior: the mean distance to 14-day neighboring territorial boundaries, and the 30-day UD of neighboring packs. Given that our Lasso procedure identified the distance to a focal pack’s own 14-day core as the top performing residential territorial term, we selected its temporally equivalent neighboring space use term for our model selection procedure (e.g., mean distance to 14-day neighboring territorial boundaries). In our RPSF analysis, our Lasso procedure identified …

S5: Averaged Resource Selection Function Covariates


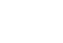

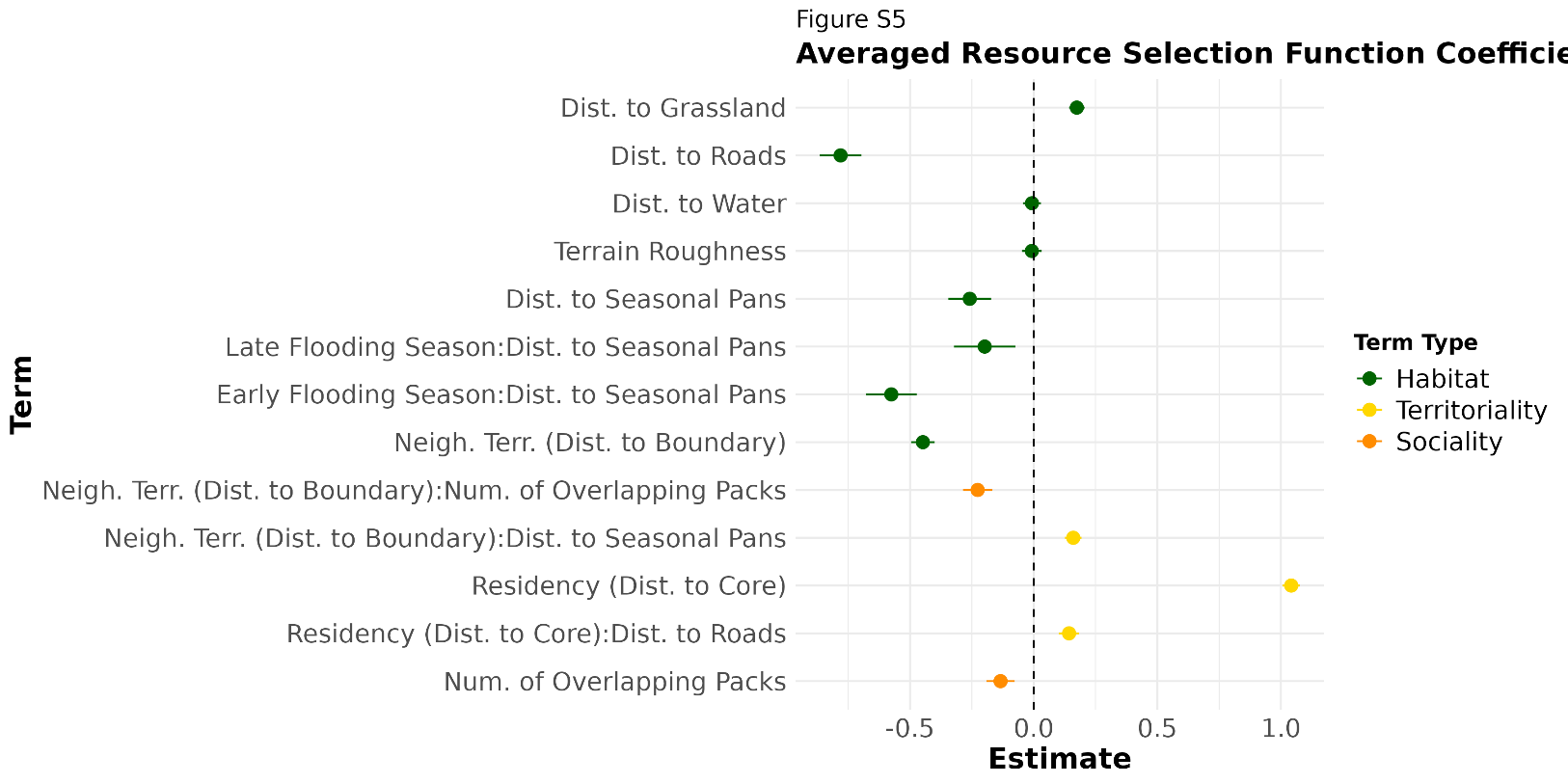


A plot depicting the covariate values of all terms included in the averaged RSF model, run in analysis 1. Terms are color-coded based on their association with either environmental features (habitat) or territorial layout (either conspecifics or residency, which is the focal pack’s territory).

S6: Averaged Resource Selection Probability (RPSF) Function Covariates


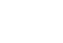

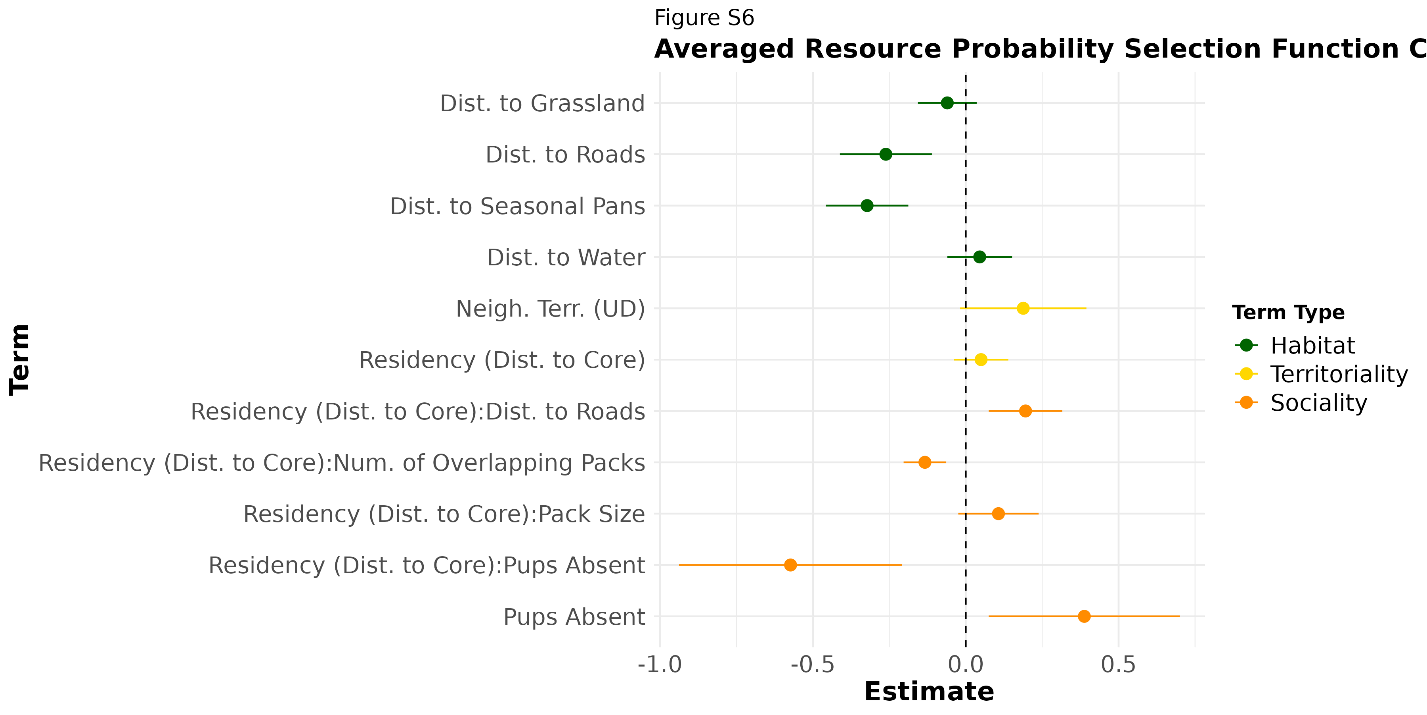


A plot depicting the covariate values of all terms included in the averaged RPSF model, run in analysis 2. Terms are color-coded based on their association with either environmental features (habitat) territorial layout (either conspecifics or residency, which is the focal pack’s territory), or social composition (such as pack size, number of overlapping packs, or presence of pups).
